# Intrinsic Motivation Mediates the Association Between Exercise-Associated Affect and Physical Activity Among Adolescents

**DOI:** 10.3389/fpsyg.2018.01151

**Published:** 2018-07-30

**Authors:** Margaret Schneider

**Affiliations:** Department of Urban Planning and Public Policy, School of Social Ecology, University of California, Irvine, Irvine, CA, United States

**Keywords:** enjoyment, youth, dual-process, health promotion, affect

## Abstract

American adolescents overwhelmingly engage in insufficient physical activity (PA). Attention has turned to the role of affect in shaping PA, raising questions as to whether the impact of affect on PA is direct/automatic or cognitively mediated (“Type 1” or “Type 2” in the dual-process model). This study examines whether intrinsic motivation (IM) mediates the association between affect and PA. Adolescents (*N* = 142, 48% Male, 20% non-Latino White, mean age = 11.04 years, mean VO2 = 37.19 ml/kg/min, mean BMI = 63.19) completed assessments of cardiorespiratory fitness, affective response to exercise on a stationary cycle, IM, preferred exercise intensity, and moderate-to-vigorous PA (MVPA; ActiGraph). Fitness, exercise intensity and MVPA assessments were repeated 5 months later. Tests for mediation showed that affect predicted PA at baseline and 5 months, and IM mediated the relationship between affect and PA both cross-sectionally (CI = 0.03, 0.17) and longitudinally (CI = 0.04, 0.18). Results suggest a cognitively mediated pathway from affect to behavior. Adolescent PA may be increased either by enhancing IM or by tailoring interventions to accommodate individuals with a predisposition to respond to exercise with negative affect. This study is registered with Clinicaltrials.gov (ID # NCT01876602).

## Introduction

Physical activity (PA) is protective against a long list of chronic diseases, including coronary heart disease, osteoporosis, diabetes, and certain forms of cancer (Booth et al., 2012). All-cause mortality is lower for active adults compared to sedentary adults even controlling for body composition (Ekelund et al., 2015). Although most of these detrimental effects of physical inactivity manifest later in life, there is increasing evidence that lack of PA also impacts the health of children and youth. There is compelling evidence that regular PA among youth leads to improved cardiorespiratory and muscular fitness, bone health, cardiovascular and metabolic health biomarkers and body composition (Physical Activity Guidelines Advisory Committee, 2008). Moreover, PA participation among school-aged children has been linked with improved brain function and academic performance (Castelli et al., 2015). There is no doubt, therefore, that an active lifestyle carries considerable benefits for the health and well-being of children and adolescents.

Unfortunately, rates of PA participation among youth are distressingly low, are reflected in poor performance on cardiorespiratory fitness tests, and generally decline during the adolescent years. Recent data from the National Health and Nutrition Examination Survey (NHANES) indicate that in 2012 only about 25% of United States youth aged 12–15 years met the current recommendation for at least 60 min per day of moderate-to-vigorous physical activity (MVPA) (Fakhouri et al., 2014). In corresponding data from the NHANES National Youth Fitness Survey, less than half (42%) of youth aged 12–15 years had adequate levels of cardiorespiratory fitness, based on standards for age and sex (Gahche et al., 2014). Data from the 2015 Youth Risk Behavior Survey (Kann et al., 2016) indicate that 53.7% of 9^th^-grade students reported engaging in 60 min of MVPA on at least five out of the last 7 days, compared to 43.5% of 12^th^-grade students. These data are consistent with a pooled analysis of 26 longitudinal studies that concluded that PA participation among 12–15 year-olds declined by 5.9% each year (Dumith et al., 2011). Overall, the data clearly indicate that United States youth are at risk as a consequence of inadequate PA participation.

Considerable research has been devoted to studying why some people engage in regular PA and others do not, yet a recent “report card” on PA among children and youth in the United States (National Physical Activity Plan Alliance, 2016) assigned the nation a D- for meeting goals for overall PA, and the Mid-Course Report on the 2007 PA Guidelines (USDHHS, 2012) called for additional research to identify effective interventions to promote youth PA. The 1996 Surgeon General’s Report on PA and Health (Centers for Disease Control and Prevention, 1996) offered a comprehensive summary of the theories and models that had up until that time largely informed efforts to impact PA behavior. Conspicuously absent from this list was the role of individual affect. In recent years, especially the last decade, a growing chorus of researchers has been arguing that one of the reasons we have been unable to develop effective strategies of PA promotion is that we have paid inadequate attention to the affective experience of exercise (Williams, 2008; Rhodes et al., 2009; Ekkekakis and Dafermos, 2012).

Affect has been defined as “the most elementary consciously accessible affective feelings (and their neurophysiological counterparts) that need not be directed at anything” (Russell and Barrett, 1999, p. 806). Typically differentiated on the basis of valence (i.e., good/bad), affect has been proposed to impact behavior either indirectly, as a result of cognitive processing (Baumeister et al., 2007), or directly, by informing an implicit attitude toward the behavior, resulting in affectively-driven motivation (Williams and Evans, 2014). The present study tests the indirect pathway leading from the immediate affective response via intrinsic motivation (IM) through to measured activity levels.

To demonstrate an indirect pathway from affect to PA participation, it is necessary to show that: (1) exercise-associated affect is related to PA participation; (2) exercise cognitions (i.e., expectations of a particular affective experience in conjunction with future exercise) are related to PA participation; and (3) the relationship between affect and PA participation is accounted for by exercise cognitions. Supporting the first condition of mediation, a recent review of 24 studies found that a positive change in affect during moderate-intensity exercise was linked to future PA (Rhodes and Kates, 2015). Evidence for the second condition of mediation comes from a meta-analysis of 15 correlational studies and 14 interventions among youth (Nasuti and Rhodes, 2013), which found that affective judgments (defined as the overall pleasure/displeasure, enjoyment, and feeling states expected from enacting an activity or from reflection on past activity) were correlated with PA with an effect size that was larger than any other previously examined correlates of youth PA. The present study extends this earlier work by testing the third condition of mediation; namely, that exercise cognitions explain the association between exercise-associated affect and PA participation.

The specific mediator investigated in this study, IM, represents a form of behavioral motivation that is, according to Self-Determination Theory (Deci and Ryan, 1985), the most self-determined motivational orientation. An individual who is intrinsically motivated to engage in a given behavior regards that behavior as inherently pleasurable. Research with adolescents (Schneider and Kwan, 2013) shows that the affective response to moderate-intensity exercise is positively associated with IM to exercise, and adolescents’ IM for exercise has been repeatedly linked with PA participation (Owen et al., 2014). In the present study, then, IM for exercise is the intervening cognition that is explored as a potential mediator of the affect-PA association.

The studies that have been conducted into exercise-associated affect and its relationship to behavior have demonstrated that the affective response to exercise differs between individuals and that it depends on the intensity of the exercise (Schneider et al., 2009; Ekkekakis et al., 2011). In general, exercise that is at a high enough intensity to trigger anaerobic metabolic processes (i.e., those that occur in the absence of oxygen) will universally generate negative affect when sustained (Ekkekakis et al., 2008); a dynamic that preserves health and well-being by discouraging the individual from engaging in exertion that threatens homeostasis. Most studies, therefore, that have examined the relationship between affect and exercise focus on moderate-intensity exercise, which is primarily aerobic (i.e., powered by oxygen-fueled metabolic processes). Similarly, the present study focuses on the relationship of affect to moderate-intensity aerobic exercise.

The present study examines the mediation hypothesis in a sample of middle-school-aged adolescents. We hypothesized that a more positive affective response to a moderate-intensity exercise task would be associated with participation in MVPA, and that this association would be mediated by IM.

## Methods

### Procedure

Adolescents were recruited through the Physical Education (PE) program at a public middle school, using presentations and flyers distributed during the PE period. Flyers stated that volunteers must not be a member of a team sport or competing in individual sports but must be able to participate in school PE classes without restriction. Interested students provided their name and phone number to a research assistant, who then contacted parents to arrange an orientation session. All assessments were conducted on site at the school, in a classroom that had been converted to a clinical laboratory. Parent/guardians provided written consent, adolescents provided written assent, and the protocol was reviewed and approved both by the University of California Irvine Institutional Review Board and by the Research Review Committee of the Long Beach Unified School District. After completing the consent/assent process, adolescents filled out a depression inventory, and were excluded from the study if they scored at or above a moderate level of depression. Baseline assessments were conducted at least 7 days apart, in the following order: cardiorespiratory fitness testing; 7-day ActiGraph; IM; affective response to exercise; “feels good” exercise task. The 7-day ActiGraph, the cardiorespiratory fitness test, and “feels good” exercise task were repeated again approximately 5 months later. All exercise tasks were conducted on stationary cycle ergometers as these procedures have been used successfully in prior studies with adolescents (Schneider and Graham, 2009; Schneider et al., 2009; Schneider and Kwan, 2013). Study participants (*N* = 146) were recruited as part of an intervention study (Schneider et al., 2016); however, the intervention (in which adolescents were randomly assigned to receive one of two exercise prescriptions) was found to have no impact on PA.

### Measures

#### Cardiorespiratory Fitness

To obtain an estimate of cardiorespiratory fitness, participants engaged in a ramp-type progressive cycle-ergometer exercise test (Whipp et al., 1981). Participants were verbally encouraged to continue until they reached their limit of tolerance. Breath-to-breath measurement of gas exchange (ventilation, oxygen uptake, and carbon dioxide output) was viewed online and analyzed using a Sensor Medics^®^ metabolic system (Yorba Linda, CA, United States) to determine peak VO2 (both L/min and ml/kg/min). Results of the fitness test were used to calibrate the target work rate for the assessment of affective response to exercise (described below).

#### Body Mass Index (BMI)

Height (PE-AIM-101, Perspective Enterprises, Portage, MI, United States) and weight (Seca 869, Chino, CA, United States) were obtained to compute BMI (height in cm/weight in kg^2^) and BMI percentile according to the normative values provided by the Centers for Disease Control and Prevention (Centers for Disease Control and Prevention, 2012).

#### Physical Activity

Physical activity is typically quantified on the basis of frequency, duration, and intensity. In this study, one assessment of PA was employed to yield a summary index that combined all three of these components, and a second assessment focused in on the intensity dimension. Firstly, a measure of usual activity (frequency, duration, and intensity combined) was obtained using the ActiGraph (model GT3X, ActiGraph, Pensacola, FL, United States), which adolescents were requested to wear for 1 week during waking hours, with the exception of swimming or bathing. Data from the ActiGraph were analyzed using the Actilife software with the Freedson (Freedson et al., 1998) cutoff (i.e., greater than 1952 counts-per-minute) to yield the average number of minutes daily that participants engaged in MVPA. A valid day contained a minimum of eight valid hours of data. As per recommendations for obtaining a valid estimate of activity (Trost et al., 2005), a minimum of four valid days (with one weekend day) was required for a participant to be included in the analyses of average daily MVPA.

As a second independent indicator of volitional intensity of PA behavior, we recorded adolescents’ chosen work rate during a lab-based exercise task in which the instruction was to find an intensity level that “felt good.” This assessment was included on the basis of studies showing that study participants worked harder on a task when that task was linked with positive affect (Custers and Aarts, 2005). In the present study, students exercised for 30 min on a stationary cycle starting at 20% of work rate at measured peak VO2. Every 3 min, work rate (watts) was recorded and participants were invited to maintain an intensity that felt “good” by increasing or decreasing the resistance in increments of 10 watts, if desired. The specific instructions given to participants have been detailed elsewhere (Schneider, 2014). The maximum work rate chosen by the participant was used in analyses as an indicator of intensity.

#### Intrinsic Motivation

Four items from the Behavioral Regulations in Exercise Questionnaire (Mullan et al., 1997) assessed the IM to exercise. The selection of these four items was based on a factor analysis of data from 192 adolescents (Schneider and Kwan, 2013). Items were scored on a scale of 1 (not true for me) to 5 (very true for me), and the average of the four items was used as an indicator of IM. The items included on the scale (following the stem “I engage in physical activity, sports and exercise…” were: because it’s fun; because I enjoy my exercise sessions; because I find exercise an enjoyable experience; and because I get pleasure and satisfaction from participating in exercise). The four items showed high internal consistency in the present study (Cronbach’s alpha = 0.87).

#### Affective Response to Exercise

Participants completed a 30-min exercise bout at a work rate that had been calibrated in relation to the previously-completed cardiorespiratory fitness test. Students were familiarized with the cycle ergometer during the cardiorespiratory fitness test, so that the equipment was familiar by the time they engaged in this additional exercise task. The intensity was maintained at 50% of the maximum work rate reached at peak VO2, unless the participant began to show signs of fatigue, such as an inability to continue pedaling the cycle ergometer at 60–70 RPM or a heart rate above 170 BPM. A heart rate of 170 BPM corresponds to a Rating of Perceived Exertion that is “very hard” on the Borg scale (Borg, 1998), and the intent of this task was to maintain moderate-intensity exertion. In the event that the participant showed signs of fatigue, the work rate was lowered by 10 Watts so that the task could be completed. Drinking water was available and offered to the participant before, during, and after the exercise session. Throughout the task, the participant provided ratings of affect using the Feeling Scale (Hardy and Rejeski, 1989) every 3 min. The Feeling Scale is scored from -5 (very bad) to +5 (very good). Ratings were provided by pointing to a number on a printed scale, to minimize the effort and distraction caused by providing ratings during the task. A summary measure of affect was computed by averaging the Feeling Scale scores from 3 to 27 min. This approach to assessing the affective response to acute exercise addresses two measurement issues identified by Backhouse et al. (2007) that plagued many early studies in this area; namely, a prior tendency to use unvalidated measures of affect that were either overly specified (i.e., breaking affect down into such small components that the generalized affect was obscured) or were confined to one polarity of affect (i.e., positive affect versus negative affect). The method used in this study to gauge individuals’ affective response to moderate-intensity exercise has been used previously in our lab and has shown evidence of external validity among older adolescents; more positive affective responses were positively correlated with average daily time spent in MVPA as measured by accelerometry (Schneider et al., 2009). Data from a subset of participants in the present study sample provided further evidence of external validity in that affective responses were predictive of chosen work-rate on the “feels good” task but not predictive of Ratings of Perceived Exertion (Schneider and Schmalbach, 2014). Evidence for the substantive validity of the assessment within the present study sample includes a Cronbach’s alpha of 0.97 across the individual ratings of the Feeling Scale every 3 min and a positive correlation between the summary affective response score and a Feeling Scale rating provided immediately upon the conclusion of the cardiorespiratory fitness test (*r* = 0.63, *p* < 0.001).

### Data Analysis

**FIGURE 1:**
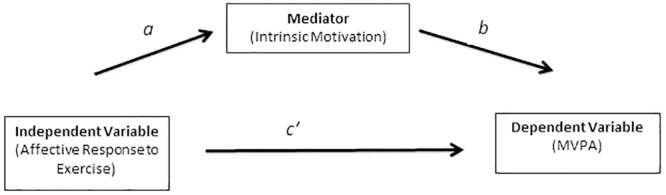
Illustration of the mediation hypothesis.

Student’s *t*-tests were utilized to examine gender differences in the main study variables. The Preacher and Hayes (2004) bootstrap mediation analysis was used to test whether IM mediated the association between affective response to exercise and PA. **Figure 1** illustrates the pattern of associations that characterizes a mediation dynamic. The Preacher and Hayes analysis tests for a relationship between the independent variable and the mediator in step one (pathway *a*). In step two, the relationship between the mediator and the dependent variable (pathway *b*) and the indirect pathway between the independent variable and the dependent variable (pathway *c′*) are evaluated simultaneously. Bootstrap mediation uses a non-parametric sampling procedure and does not assume normality of the sampling distribution of the indirect effect; therefore, it is considered to be more powerful for hypothesis testing for mediation analysis than the Sobel’s test, which assumes a normal distribution of the indirect effect. We utilized 5000 bootstrap samples for coefficient and indirect effect estimation. The indirect effect was determined to be statistically significant if the 95% bias-corrected confidence intervals (CIs) for the indirect effect did not include zero. The completely standardized effect size (CS) is reported as an index of effect size (Preacher and Kelley, 2011).

Using the Process Macro for SPSS version 23, separate analyses were run first designating MVPA as the dependent variable and then designating the maximum work rate during the “feels good” task as the dependent variable. All continuous variables representing the independent, dependent and mediator variables were centered on their mean prior to analyses. Affective response to exercise was specified as the independent variable, IM as the putative mediator, and gender and VO2 L/min covariates. All mediation analyses were repeated using a residualized form of the affective response to exercise variable which controlled for baseline affect. Because this approach did not change the results, only the results of the analyses using the raw data are presented. Analyses were conducted first using time one MVPA and work rate and then time two MVPA and work rate, essentially replicating the analyses with cross-sectional and prospective data. In the prospective analyses, both time one cardiorespiratory fitness and the change in cardiorespiratory fitness from time one to time two were included as covariates. Because gender was a significant predictor of MVPA in step two of the mediation analyses regarding MVPA (time one and time two), these analyses were repeated for each gender separately as a *post hoc* investigation into gender differences.

At baseline, two students failed to provide complete ActiGraph data, and two students withdrew from the school midway through data collection, so baseline analyses were conducted on 142 participants. At the time two assessment, complete data were available for 134 participants, so only these participants were included in the time two analyses. Because the intervention delivered to the study participants in the intervening period was determined to have no impact (Schneider et al., 2016), group assignment was not included in time two analyses.

## Results

### Study Participants

**Table 1** presents the participant characteristics of the baseline sample by gender. The mean age of participants was 11.05 years (*SD* = 0.41). On average, participants provided 6.39 (*SD* = 1.03) valid days of ActiGraph data, including a mean of 1.81 (*SD* = 0.48) weekend days. The sample was ethnically diverse, with 49% identifying as Latino, 8% Asian, 14% African–American, 20% non-Latino White, and 8% Mixed or Other. The cardiorespiratory fitness data indicate that on average these adolescents were not in the “Healthy Fitness Zone” as characterized by published standards (Welk et al., 2011), but the mean Body Mass Index percentiles indicate that the sample was generally not overweight. Consistent with general trends, boys in this study were more active and had higher cardiorespiratory fitness as compared to girls. Boys also preferred higher-intensity exercise as compared to girls.

**Table 1:** Baseline participant characteristics.

	All	Male	Female
	*n* = 142 *M (SD)*	*n* = 70 *M (SD)*	*n* = 72 *M (SD)*
BMI (cm/kg^2^)	20.41 (4.54)	20.63 (4.67)	20.08 (4.43)
BMI percentile	63.19 (31.49)	65.44 (30.74)	59.91 (32.53)
Peak VO2 (L/min)^∗∗^	1.64 (0.31)	1.73 (0.32)	1.56 (0.28)
Peak VO2 (ml/kg/min)^∗∗^	37.19 (7.48)	38.98 (7.69)	35.64 (6.89)
MVPA (min/day)^∗∗∗^	46.00 (20.10)	53.13 (22.28)	39.71 (14.95)
Chosen max work rate (Watts)^∗^	33.87 (13.42)	36.42 (14.32)	31.38 (12.08)
Intrinsic motivation	5.68 (1.27)	5.73 (1.37)	5.63 (1.18)
Affective response	2.73 (1.83)	2.63 (1.90)	2.77 (1.87)

*Gender differences, evaluated using *t*-tests: ^∗^*p* < 0.05; ^∗∗^*p* < 0.01; ^∗∗∗^*p* < 0.001.*

### Test for Mediation

#### Moderate-to-Vigorous Physical Activity

The test of simple mediation showed that the affective response to exercise had a significant effect on time one MVPA that was mediated by IM (see **Table 2**). There was an effect of affective response to exercise on IM (β = 0.25, *SE* = 0.05, *t* = 4.60, *p* < 0.001), and an effect of IM on time one MVPA (β = 3.81, *SE* = 1.28, *t* = 2.97, *p* < 0.01). As the 95% confidence interval for the indirect effect (CI = 0.03, 0.17) did not include zero, mediation was confirmed. The completely standardized effect size for the indirect effect (*CS* = 0.09) suggests a small effect according to Cohen’s conventions. Overall, the predictors explained 22% of the variance in time one MVPA.

**Table 2:** Results of the cross-sectional test for mediation predicting MVPA (*N* = 142).

				5000 sample bootstrapping	
Predictors	*b* (SE)	*t* Score	*p*-value	*b* (SE)	95% BCa CI
**Step One (pathway *a*); Dependent Variable = Intrinsic Motivation**					
Constant	0.03 (0.32)	0.09	0.92		
Gender	–0.20 (0.20)	–0.14	0.88		
VO2 L/min	0.79 (0.32)	2.43	0.01		
FS	0.25 (0.05)	4.60	0.0000		
**Step Two (paths *b* and *c′*); Dependent Variable = Time One MVPA**					
Constant	18.40 (4.90)	3.75	0.000		
Gender	–12.36 (3.09)	–3.99	0.000		
VO2 L/min	9.05 (5.01)	1.80	0.07		
FS	0.60 (0.88)	0.68	0.49		
IM	3.81 (1.28)	2.97	0.003		
Indirect effect				0.09 (0.03)	[0.038, 0.177]

*VO2 L/min, cardiorespiratory fitness; FS, affective response to exercise; IM, intrinsic motivation; MVPA, minutes per day of moderate-to-vigorous physical activity.*

Similar results were obtained in analyses using the time two MVPA as the outcome variable (see **Table 3**). There was an effect of affective response to exercise on IM (β = 0.26, *SE* = 0.05, *t* = 4.64, *p* < 0.001), and an effect of IM on time two MVPA (β = 4.39, *SE* = 1.53, *t* = 2.86, *p* < 0.01). As the 95% confidence interval for the indirect effect (CI = 0.04, 0.18) did not include zero, mediation was confirmed. The completely standardized effect size for the indirect effect (CS = 0.09) suggests a small effect according to Cohen’s conventions. Overall, the predictors explained 24% of the variance in time two MVPA.

**Table 3:** Results of the prospective test for mediation predicting MVPA 5 months later (*N* = 134).

				5000 sample bootstrapping	
Variable	*b* (SE)	*t* Score	*p*-value	*b* (SE)	95% BCa CI
**Step One (pathway *a*); Dependent Variable = Intrinsic Motivation**					
Constant	–0.02 (0.33)	–0.06	0.94		
Gender	0.00 (0.21)	0.03	0.97		
VO2 L/min	0.78 (0.34)	2.29	0.02		
VO2 L/min change	0.54 (0.44)	1.20	0.22		
FS	0.26 (0.05)	4.64	0.0000		
**Step Two (paths *b* and *c′*); Dependent Variable = Time Two MVPA**					
Constant	26.66 (5.75)	4.62	0.00		
Gender	–17.64 (3.66)	–4.81	0.0000		
VO2 L/min	6.19 (6.10)	1.01	0.31		
VO2 L/min change	9.56 (7.84)	1.22	0.22		
FS	0.01 (1.08)	0.01	0.99		
IM	4.39 (1.53)	2.86	0.00		
Indirect effect				0.10 (0.03)	[0.041, 0.184]

*VO2 L/min, cardiorespiratory fitness; FS, affective response to exercise; IM, intrinsic motivation; MVPA, minutes per day of moderate-to-vigorous physical activity.*

#### Work Rate

The test of mediation in the association between affect and preferred work rate showed that the association between affective response to exercise and maximum work rate during the time one “feels good” task was not mediated by IM (see **Table 4**). Although the independent variable (affect) was associated with the putative mediator (IM) in step one, and the association between IM and work rate was trending toward significance in step two, the 95% confidence interval for the indirect effect (CI = 0.000, 0.143) did include zero. Therefore, according to the criteria for mediation, mediation was not confirmed.

**Table 4:** Results of the cross-sectional test for mediation predicting Work Rate (Watts) (*N* = 142).

				5000 sample bootstrapping	
Variable	*b* (SE)	*t* Score	*p*-Value	*b* (SE)	95% BCa CI
**Step One (pathway *a*); Dependent Variable = Intrinsic Motivation**					
Constant	0.03 (0.32)	0.09	0.92		
Gender	–0.02 (0.20)	–0.14	0.88		
VO2 L/min	0.79 (0.32)	2.43	0.01		
FS	0.25 (0.05)	4.60	0.0000		
**Step Two (paths *b* and *c′*); Dependent Variable = Time One work rate**					
Constant	3.16 (3.16)	0.99	0.31		
Gender	–2.20 (1.99)	–1.10	0.27		
VO2 L/min	18.36 (3.23)	5.67	0.0000		
FS	1.12 (0.56)	1.97	0.05		
IM	1.49 (0.82)	1.79	0.07		
Indirect effect				0.05 (0.03)	[0.000, 0.143]

*VO2 L/min, cardiorespiratory fitness; FS, affective response to exercise; IM, intrinsic motivation.*

The findings were different when using the time two work rate data (see **Table 5**). There was an effect of affective response to exercise on IM (β = 0.26, *SE* = 0.05, *t* = 4.64, *p* < 0.001), and an effect of IM on time two work rate (β = 1.85, *SE* = 0.88, *t* = 2.10, *p* < 0.05). As the 95% confidence interval for the indirect effect (CI = 0.008, 0.173) did not include zero, mediation was confirmed. The completely standardized effect size for the indirect effect (CS = 0.07) suggests a small effect according to Cohen’s conventions. Overall, the predictors explained 22% of the variance in time two work rate.

**Table 5:** Results of the prospective test for mediation predicting Work Rate (Watts) 5 months later (*N* = 134).

				5000 sample bootstrapping	
Variable	*b* (SE)	*t* Score	*p*-value	*b* (SE)	95% BCa CI
**Step One (pathway *a*); Dependent Variable = Intrinsic Motivation**			
Constant	–0.02 (0.33)	–0.06	0.94		
Gender	0.00 (0.21)	0.03	0.97		
VO2 L/min	0.78 (0.34)	2.29	0.02		
VO2 L/min change	0.54 (0.44)	1.20	0.22		
FS	0.26 (0.05)	4.64	0.0000		
**Step Two (paths *b* and *c′*); Dependent Variable = Time Two work rate**			
Constant	4.95 (3.30)	1.49	0.13		
Gender	–3.40 (2.10)	–1.62	0.10		
VO2 L/min	13.27 (3.50)	3.78	0.0002		
VO2 L/min change	9.30 (4.50)	2.06	0.04		
FS	0.80 (0.62)	1.29	0.19		
IM	1.85 (0.88)	2.10	0.03		
Indirect effect				0.07 (0.04)	[0.008, 0.173]

*VO2 L/min, cardiorespiratory fitness; FS, affective response to exercise; IM, intrinsic motivation.*

#### Gender Differences

Because gender was significantly associated with MVPA in the second step of the mediation test, a *post hoc* analysis was conducted to examine whether the mediation was operating consistently for both boys and girls. Results of this analysis revealed that the mediation dynamic was upheld among boys, but not among girls. Among girls, affect was significantly associated with IM, but in the second step of the analyses there was no significant association between IM and MVPA. Moreover, the confidence interval for the indirect effect contained zero. Thus, among girls there was no indirect association between affect and MVPA at time one or time two.

## Discussion

This analysis of data from 142 adolescent boys and girls supported the hypothesis that IM to exercise mediates the association between the affective response to acute exercise and PA behavior; however, the mediation emerged only among boys and was not supported among girls. The strength of the evidence from this study is bolstered by the rigorous methods employed; namely, objective assessments of the affective response to exercise and PA. Moreover, we found that the affective dimensions of exercise had implications not only for the quantity of volitional activity (i.e., average daily MVPA) but also for the intensity of exercise (i.e., chosen work rate during the “feels good” exercise task).

It is worth noting that the gender differences in the results and the relatively small amount of variance in behavior explained (i.e., less than 25%) together suggest that there are substantial additional factors influencing PA participation that are external to the model tested in the present study. The social ecological model of PA (Sallis et al., 2006) posits that behavior is influenced by both individual-level characteristics, such as gender, and by multiple dimensions of the context within which the behavior takes place, including family, peers, and physical environment. Evidence for this model comes from studies showing that some portion of PA participation among adolescents may be attributed to socioeconomic status (Elgar et al., 2015), perceived neighborhood safety (Cote-Lussier et al., 2015), and/or proximity of recreational resources (Carroll-Scott et al., 2013). Moreover, there may be cross-level interactions, for example between gender and environmental factors, that generate disparities in activity across gender. That girls participate in less PA than boys across childhood and adolescence has been thoroughly documented (Eaton et al., 2008; Whitt-Glover et al., 2009). Data from the Trial of Activity for Adolescent Girls indicate that among girls PA barriers are consistently associated with MVPA between 6^th^ and 11^th^ grades (Young et al., 2014). Together with our findings, these data suggest that girls’ participation in PA may be hindered by factors outside the intrapersonal sphere, and the impact of these factors may diminish the role that exercise-associated affect and IM play in shaping girls’ daily MVPA.

The present findings are consistent with the feedback hypothesis described by Baumeister et al. (2007), wherein they argue that “the main direct impact of emotion is to stimulate cognitive processing, not behavior (p. 174).” Essentially, this hypothesis posits that affect prompts a learning process whereby the individual incorporates the anticipated affect associated with a given behavior into decisions that guide future behavior. Self-Determination Theory (Ryan and Deci, 2017), which includes IM as a significant driver of behavior, may thus account for the influence of affect on behavior without the need to directly account for the original affective experience that informs IM. Nevertheless, being able to predict or explain a behavior does not imply that a pathway to change behavior will flow from this understanding. That is, knowing that IM predicts behavior is useful, but identifying the factors that shape IM is necessary to leverage understanding into behavior change. Accordingly, the present finding that the affective experience associated with acute exercise influences PA by modifying IM offers clear implications for intervening to promote increased levels of PA.

The implications of these findings for the effective promotion of physically active lifestyles among youth depends heavily on whether the affective response to exercise is stable, reflecting an underlying characteristic or trait, or subject to modification through experience. If indeed the affective response to exercise is subject to modification through experience, our findings would suggest that future research should seek to identify strategies for improving individuals’ affective experience during exercise. There have in fact been a number of studies showing that exercise enjoyment can be enhanced acutely through the use of music (Karageorghis et al., 2012), the natural environment (Thompson Coon et al., 2011), exercise intensity (Ekkekakis et al., 2005), or nutritional manipulation (Schubert et al., 2014). Studies have not, however, documented whether repeated exposures to conditions that promote positive affect during exercise will over time modify the individuals’ affective response to a standardized exercise stimulus.

Limitations to this study include the exclusion criteria used in recruitment (i.e., no participation in team or individual competitive sport), the potential error inherent in the measure of MVPA, and the omission of contextual variables and measures of other forms of self-regulation in the analyses. Adolescents who were active in competitive sports (group or individual) were excluded from the study; therefore, the results may not be generalizable to athletic students. The use of the ActiGraph to measure MVPA has the advantage of not being subject to the weaknesses of self-report, but does represent a “snapshot” of activity in a single week. This week may or may not be representative of a typical week for a particular child. There are other forms of self-regulation described in Self-Determination Theory (i.e., external and introjected regulation) that were not included in this study; while not expected to be related to IM, these forms of self-regulation may explain some of the variance in MVPA. Finally, this study took a reductionist approach to the prediction of PA, and did not include additional social-cognitive variables (e.g., perceived competence, autonomy, and relatedness) and/or environmental variables (e.g., neighborhood safety) that may influence PA. However, the strengths of the study include the rigorous measures of affect and PA as well as the replication of findings both cross-sectionally and longitudinally.

There are additional characteristics of the particular exercise task used to assess affective response to exercise that should be considered when interpreting the study findings. The task utilized was indoor stationary cycling, which may not elicit the same affective response as an outdoor activity or a different mode of activity might elicit (e.g., running). The intent behind the standardized task was to tap into the psychophysiological impact of exercise *per se* on each adolescent, divorced from situational factors that clearly may influence affective response, such as music, social interaction, competition, or a natural setting. This approach is predicated on the assumption that each individual brings to the experience of exercising an inherent predisposition to experience the physical demands as pleasant or unpleasant. Future studies could build on these findings by examining whether IM would mediate the effect of affective response to exercise when the exercise task is more ecologically valid.

The findings of this study suggest that individuals who experience a positive affective response to acute exercise are “primed” to be intrinsically motivated to exercise, and might be encouraged to exercise by removing the barriers to the behavior. In contrast, those who experience a negative affective response to acute exercise may simply not find exercise pleasurable and therefore are in need of a completely different strategy. Possible strategies might include linking exercise to necessary or preferred activities (e.g., walking to work or school; walking with a friend or family member), encouraging acceptance of the negative aspects of exercise while focusing on long-term benefits, or offering extrinsic rewards for being physically active (e.g., social approbation through social media, financial rewards through employee health programs). What is unlikely to work with these reluctant exercisers is any attempt to convince them that exercise in and of itself is “fun.” Thus, the ultimate value of the present findings may be in guiding the effective allocation of resources toward the right targets to ultimately promote PA throughout the whole population.

### Future Studies

There is some evidence that suggests that the fundamental affective response to a standardized exercise stimulus, in the absence of environmental manipulations, may be relatively stable. A recent article (Schutte et al., 2017) examining the affective response to exercise among monozygotic and dizygotic twins found that the affective response to exercise had a heritable genetic component, with genetic factors explaining 15% of individual differences in Feeling Scale responses during a cycle ergometer task. There are grounds, therefore, for positing that at least some portion of the affective response to acute exercise may be a stable trait.

Assuming that the affective response to acute exercise is to some degree a stable trait, the current study findings may be most useful when interpreted in conjunction with recent studies into the role of cognitive acceptance and behavioral commitment in the promotion of PA (Butryn et al., 2015, 2016). This approach draws upon the field of mindfulness, and sets forth the possibility that behavior change that is not inherently rewarding may still be successfully accomplished if the individual learns to accept the unpleasant aspects of the behavior and focus attention on values or long-term goals that support the behavior. Conceptually, there is overlap between acceptance-based approaches and Self-Determination Theory (Ryan and Deci, 2017), which identifies IM as the most powerful influence promoting a behavior but acknowledges as well the potential effectiveness of introjected regulation, “a form of control that people enact on themselves, emphasizing internal judgments and evaluations upon which feelings of worth are conditional (Ryan and Deci, 2017, p. 185).” Future studies should explore whether acceptance-based interventions can be effective for promoting PA participation among individuals with low IM to exercise.

## Author Contributions

MS conceived of the study, supervised the acquisition of the data, analyzed the data, and drafted the manuscript.

## Conflict of Interest Statement

The author declares that the research was conducted in the absence of any commercial or financial relationships that could be construed as a potential conflict of interest.
